# Decay of homologous chromosome pairs and discovery of males in the thelytokous fungus-growing ant *Mycocepurus smithii*

**DOI:** 10.1038/s41598-022-08537-x

**Published:** 2022-03-22

**Authors:** Luísa Antônia Campos Barros, Christian Rabeling, Gisele Amaro Teixeira, Cléa dos Santos Ferreira Mariano, Jacques Hubert Charles Delabie, Hilton Jeferson Alves Cardoso de Aguiar

**Affiliations:** 1 Universidade Federal do Amapá, Campus Binacional, Oiapoque, Amapá Brazil; 2 School of Life Sciences, Arizona State University, Tempe, AZ USA; 3 Programa de Pós-Graduação em Biologia Celular e Estrutural, Departamento de Biologia Geral, Universidade Federal de Viçosa, Viçosa, Minas Gerais Brazil; 4 Departamento de Ciências Biológicas, Universidade Estadual de Santa Cruz, Ilhéus, Bahia Brazil; 5 Departamento de Ciências Agrárias e Ambientais, Universidade Estadual de Santa Cruz, Ilhéus, Bahia Brazil; 6 Programa de Pós-Graduação em Biodiversidade Tropical, Universidade Federal do Amapá, Campus Marco Zero, Macapá, Amapá Brazil

**Keywords:** Cytogenetics, Entomology, Mitosis, Evolutionary biology, Chromosomes, Meiosis

## Abstract

The prevalent mode of reproduction among ants is arrhenotokous parthenogenesis where unfertilized eggs give rise to haploid males and fertilized eggs develop into diploid females. Some ant species are capable of thelytokous parthenogenesis, a type of asexual reproduction where females develop from unfertilized diploid eggs. Thelytoky is well-documented in more than 20 ant species. Cytogenetic data are available for six species demonstrating that some thelytokous ant species are capable of producing males occasionally as well as maintaining their chromosome numbers and proper chromosome pairings. *Mycocepurus smithii* is a thelytokous fungus-growing ant species that inhabits large parts of Central and South America. Cytogenetic data are unavailable for *M. smithii* and male individuals were never documented for this species, although the presence of males is expected because genetic recombination was observed in a few sexually reproducing populations in Brazil and haploid sperm was documented from the spermathecae of *M. smithii* queens. This study aims at comparatively studying asexual and sexual populations of *M. smithii* using classical and molecular cytogenetic methods to test whether karyotype configuration is modified according to the mode of reproduction in *M. smithii*. Moreover, we report the discovery of *M. smithii* males from a sexually reproducing population in the Brazilian state Pará, diagnose the male of *M. smithii*, and morphologically characterize their spermatozoa. Karyotypic variation was observed within the asexual population (2n = 9, 10, or 11), whereas the chromosome number was fixed in the sexual population (2n = 14, n = 7). Identical karyotypes were maintained within individual *M. smithii* colonies and karyotype variation was only observed between colonies. In asexual individuals, the karyomorphs showed a decay of homologous chromosome pairs, especially in individuals with the karyomorph 2n = 11, which is potentially caused by relaxed natural selection on proper chromosome pairing. In contrast, females in the sexual population showed proper homologous chromosome pairings. In individuals of both asexual and sexual populations, we find that heterochromatin was localized in centromeric regions and on the short arms of the chromosomes, GC-rich regions were associated with heterochromatic regions, and 18S rDNA genes were located on the largest chromosome pair. This comparative cytogenetic analysis contributes to our understanding about the cytological mechanisms associated with thelytokous parthenogenesis in ants and suggests the decay of chromosome structure in the absence of meiosis and genetic recombination.

## Introduction

The ants, wasps, and bees comprise the hyper-diverse insect order Hymenoptera that originated during the Triassic and consists of at least 165,000 extant species with many more species awaiting discovery^1^. Although eusociality evolved repeatedly across the tree of life, the Hymenoptera are unique in that eusociality evolved more than ten times independently in this group^2^. Hymenoptera are also remarkable in that arrhenotokous parthenogenesis is part of their regular life cycle, while it is rare or absent from other arthropods, except for scale insects and mites^3–5^. In arrhenotokous organisms, males are haploid and develop from unfertilized eggs, whereas females are diploid and develop from fertilized eggs. In contrast, thelytoky, or thelytokous parthenogenesis, is a form of asexual reproduction where females develop from unfertilized diploid eggs^5–10^. Thelytoky evolved secondarily from arrhenotoky in Hymenoptera^11^ and is considered relatively rare among eusocial Hymenoptera. Notwithstanding, well-documented reports for at least twenty thelytokous ant species exist^10,12–15^. The high diversity of reproductive modes and the underlying genetic mechanisms were suggested to play an important role in the repeated evolution of thelytoky in eusocial Hymenoptera^10,16^.

In Hymenoptera, two types of thelytoky are known: apomixis and automixis^5–10^. In apomixis, or mitotic parthenogenesis, meiosis is absent and the cytological mechanism is usually simple without homologous chromosome pairings and without fusion of meiotic products. Therefore, the offspring are genetically identical clones of their mothers. The existing heterozygosities and newly arisen variation from mutation are perpetuated from generation to generation. In automixis, or meiotic parthenogenesis, functional meiosis occurs with pairings of homologous chromosomes and is followed by a reduction of sister chromatids. Diploidy is restored via the fusion of the polar nuclei (central fusion), the fusion of polar nuclei with the egg nucleus (terminal fusion), or via gamete duplication. Each cytological mechanism has different consequences for the genetic composition of the progeny^10,17^. The absence or reduced frequency of recombination in asexual populations bear consequences for the morphology of chromosomes because non-homologous chromosome pairings can result in mutations that would be deleterious in arrhenotokous organisms^6,7^.

Among the fungus-growing ants, *Mycocepurus smithii* (Forel, 1893) is the only known species that reproduces via thelytokous parthenogenesis^18–21^. The population structure of *M. smithii* was investigated using 12 variable microsatellite loci across the species’ biogeographic distribution from Mexico to Argentina showing that *M. smithii* constitutes a mosaic of sexually and asexually reproducing populations^21^. The sexually reproducing *M. smithii* populations were found in the center of the species distribution range in the Amazonas basin of Brazil, whereas asexual populations occurred across Latin America as far as Mexico to the north and as far as Argentina to the south^21^. The presence of sperm in the spermathecae of queens in recombinant, sexually reproducing *M. smithii* populations also indicated the presence of males^21^, however, male individuals of *M. smithii* were never discovered until this study^10,18–23^. Molecular phylogenetic analyses of the genus *Mycocepurus* suggests that *M. smithii* is monophyletic and closely related to a sexually reproducing species native to South America^21,24^. On the population level, neither the sexual nor the asexual populations were monophyletic, which is consistent with the hypothesis that asexuality evolved repeatedly and independently in *M. smithii*. The origin of thelytoky was inferred to be recent, approximately 0.5–1.65 million years ago^21^.

To date, cytogenetic analyses are absent for any population of *M. smithii* and the males of *M. smithii* are only indirectly known from sperm found in spermathecae of *M. smithii* queens. Here, we comparatively study the cytogenetic condition of asexually and sexually reproducing *M. smithii* populations to understand the cytogenetic consequences of asexual reproduction on chromosome formation and further investigate the cytogenetic mechanism responsible for thelytoky in *M. smithii*. Furthermore, we discovered the males of *M. smithii.* We here taxonomically diagnose these males and describe their sperm morphology. We discuss our findings in the context of the cytological consequences that asexual reproduction has on chromosome evolution in asexual ants.

## Materials and methods

### Population sampling

Cytogenetic analyses were conducted for a total of 149 individuals of *M. smithii* with 116 individuals from 49 asexual colonies and 33 individuals from eleven sexually reproducing colonies. The asexually reproducing colonies were collected at two localities in the state of Minas Gerais in Brazil between March 2010 and December 2012 (Table 1). Seven colonies were collected in Viçosa (20° 41′ S; 42° 54′ W) and 42 colonies were collected in Ponte Nova (20° 45′ S 42° 52′ W). The two collection localities were approximately 60 km apart. The eleven sexually reproducing colonies of *M. smithii*, all of which contained males, were collected in Belém in the state of Pará in Brazil (1° 27′ S 48° 26′ W) during October of 2012.

**Table 1:** Colonies of *Mycocepurus smithii* studied for the cytogenetic comparison. Information about the mode of reproduction, collection locality of the sample, number of colonies examined, number of workers, gynes and males analyzed via classical and molecular cytogenetic methods, diploid (2n) and haploid (n) chromosome numbers, and karyotypic formula are presented.

Mode of reproduction	Collection locality	# of colonies examined	# of workers, [*gynes*], (males) studied	Chromosome number 2n; (n)	Karyotypic formula 2n; (n)
Asexual	Viçosa, Minas Gerais, Brazil	**1**	3	10	2m + 7sm + 1st
	Viçosa, Minas Gerais, Brazil	**6**	13	11	2m + 6sm + 2st + 1a
	Ponte Nova, Minas Gerais, Brazil	**12**	24, [9]	9	1m + 7sm + 1st
	Ponte Nova, Minas Gerais, Brazil	**2**	4	10	2m + 7sm + 1st
	Ponte Nova, Minas Gerais, Brazil	**28**	55, [8]	11	2m + 6sm + 2st + 1a
Sexual	Belém, Pará, Brazil	**11**	27, (14)	14; (7)	4m + 6sm + 2st + 2a; (2m + 3sm + 1st + 1a)

Colonies of the asexual *M. smithii* population from Minas Gerais were kept alive in the Laboratório de Citogenética de Insetos at the Universidade Federal de Viçosa for six months (24/VI/2012 until 30/XII/2012) to obtain larvae for cytogenetics and to test whether males could be raised from asexual colonies. Males were not produced by the asexual colonies from Minas Gerais although the production of winged queens (gynes) occurred during that period. Colonies of the arrhenotokous attine ant *Mycetomoellerius relictus* (Borgmeier, 1934) were also kept in the laboratory during the same period and, unlike *M. smithii*, produced males and gynes, suggesting that the laboratory conditions were probably adequate for keeping fungus-growing ants. Colonies were maintained in plastic containers that were sealed with perforated lids to enable gas exchange and covered with moist cotton to increase humidity in artificial laboratory nests. Collections were authorized by the Instituto Chico Mendes de Conservação da Biodiversidade (ICMBio) (SISBIO accession number 32459) issued to Luísa Antônia Campos Barros. Specimens were identified to species by Jacques Hubert Charles Delabie and Christian Rabeling. Vouchers were deposited in the myrmecological collections of the Centro de Pesquisas do Cacau (CPDC) at the Comissão Executiva do Plano da Lavoura Cacaueira (CEPLAC), in Bahia, Brazil, records #5706 and #5707 for Viçosa and Ponte Nova, respectively, and under the record #5714 for specimens from Belém, as well as in the Social Insect Biodiversity Repository at the School of Life Sciences at Arizona State University in Tempe, Arizona, USA.

### Chromosome preparation, banding, and karyotype analyses

Mitotic metaphase chromosomes were obtained following the protocol developed by Imai et al*.*^25^. For the asexual individuals, brain ganglia of prepupae, which are recognizable by the presence of a visible head, and post-defecating larvae of workers and gynes were analyzed. For the sexual individuals, larval brain ganglia of workers, gynes, and males, in addition to testes of pupae and larvae were used. To characterize the karyotypes of *M. smithii* females, the larvae and prepupae of workers and gynes were first confirmed as females as evident by the absence of testes. We then distinguished worker from queen prepupae. Queen prepupae had distinctly larger body sizes and head widths than worker prepupae. Chromosome numbers and chromosome morphologies were identified using the Giemsa staining 4%.

Chromosomes were measured and arranged in order of decreasing size, and based on the ratios of chromosome arm lengths (r = long arm/short arm) according to the classification proposed by Levan et al. ^26^. The chromosomes were classified as m = metacentric (r = 1–1.7), sm = submetacentric (r = 1.7–3), st = subtelocentric (r = 3–7), and a = acrocentric (r > 7). Additionally, the karyotype assembly was conducted by pairing the probable chromosomes which suffered rearrangements. Images were edited using Adobe Photoshop version 23.2.0.

The heterochromatin regions were detected using Giemsa staining 4% according to Imai^27,28^ and also according to Sumner^29^, with modifications proposed by Barros et al. ^30^. Metaphasic chromosomes were stained with the sequential fluorochromes CMA_3_/DA/DAPI to detect GC and AT-rich regions based on the methodology proposed by Schweizer ^31^.

The major rDNA genes, i.e., tandemly repeated arrays of genes constituting nucleolus organizing regions (NORs), were detected via fluorescence hybridization (FISH), following the protocol of Pinkel et al.^32^ with the use of the 18S rDNA probes by amplification via polymerase chain reaction (PCR). The probes were constructed using *Melipona quinquefasciata* Lepeletier, 1836, rDNA primers 18SF1 (5′-GTC ATA GCT TTG TCT CAA AGA-3ʹ) and 18SR1.1 (5′-CGC AAA TGA AAC TTT AAT CT-3ʹ)^33^ in the genomic DNA of the ant *Camponotus rufipes* (Fabricius, 1775). Gene amplification was performed following Pereira^33^. The 18S rDNA probes were labeled by an indirect method using digoxigenin-11-dUTP (Roche Applied Science, Mannheim, Germany), and the FISH signals were detected with anti-digoxigenin-rhodamine (Roche Applied Science), following the manufacturers’ protocols.

The metaphase chromosomes were observed and documented using an Olympus BX 60 fluorescence microscope with a Q-Color3 Olympus® image capturing system, using the software Q capture^®^ with the filters WB (450–480 nm), WU (330–385 nm) and WG (510–550 nm) for analyzing CMA_3_, DAPI, and rhodamine, respectively.

### Morphological characteristics of ovaries and sperm

Ten asexually reproducing queens, each one from a different laboratory colony, were dissected to study ovary morphology and development. All individuals were dissected in buffered Ringer's solution to avoid desiccation of soft morphological structures. Female reproductive parts were studied using an Olympus SZ40 stereo microscope. We examined whether queens were inseminated (empty/translucent versus filled/opaque spermatheca), and whether they were reproductively active as evident by the presence of yellow bodies (*Corpora lutea*) and fully developed oocytes^19–21^. Asexual, reproductively active females were expected to have empty spermathecae, fully developed ovaries with mature oocytes, and *corpora lutea* as indicators of past oviposition. Asexual females that were never reproductively active were expected to have empty spermathecae, as well as ovaries with undeveloped oocytes and without *corpora lutea.* In contrast, sexually reproducing females were expected to have sperm filled spermathecae, developed oocytes, and *corpora lutea*. However, the reproductive tract of immature sexual and asexual queens could be identical if they were dissected pre-mating and/or pre-reproduction, or if sexually reproducing individuals were dissected post-mating and pre-reproduction, they would be inseminated with developing ovaries but without *Corpora lutea*^20^.

To study the male reproductive morphology, seminal vesicles of three males from a single colony were dissected on histological slides in phosphate buffered saline solution (pH 7.2) and, then fixed in 4% paraformaldehyde and 0.1 M phosphate buffer for 20 min. The spermatozoa were stained with 4,6-diamidino-2-phenylindole (0.6 µg/mL) for 30 min in McIlvaine buffer (pH 7.0) and then washed using the same buffer. Finally, the slides were wet mounted with 50% sucrose and covered with cover slides for nucleus measurements. The nuclei were observed and photographed using an Olympus BX 60 fluorescence microscope with a Q-Color3 Olympus^®^ image capture system, using the software Q capture^®^ with the filter WU (330–385 nm). After the removal of the fluorochrome, slides were stained with Giemsa 4% for 20 min and photographed using an Olympus BX 60 light microscope to obtain the total length of the sperm and their nuclei. A total of 20 randomly selected spermatozoa were measured per male using the software package Image Pro Plus^®^.

### Taxonomic characterization of the *Mycocepurus smithii* males

*Mycocepurus* individuals were examined and measured using a Leica M205 C stereomicroscope fitted with an ocular micrometer. Measurements were taken at 50× and 63× magnification and recorded to the nearest 0.01 mm at the maximum magnification allowed for each measurement without exceeding the bounds of the micrometer. Composite images were generated using a Leica DFC450 digital camera mounted to a Leica M205 C stereomicroscope and assembled using Leica Application Suite (version 4.5) and Helicon Focus (version 6.6.1) software packages. Morphological terminology, measurements and indices used for the taxonomic description follow recent taxonomic studies of fungus-growing ants^34,35^. Measurements are given in millimeters. Abbreviations for measurements and indices are as follows:

**Cephalic index (CI)**: HW/HL × 100.

**Eye length (EL)**: Maximum diameter of the eye from the dorsal to the ventral margin, measured in full-face view.

**Head length (HL)**: Maximum vertical distance of the head in full-face view, excluding mandibles, measured in a straight line from the midpoint of the anterior clypeal margin to the midpoint of the posterior margin of the head. In species where the posterior margin of the head or the clypeal margin (or both) is concave, the measurement is taken from the midpoint of a transverse line spanning the anteriormost or posteriormost projecting points, respectively.

**Head width (HW)**: Maximum horizontal width of the cephalic capsule, excluding the eyes, measured in full-face view.

**Petiole length (PL)**: Maximum length of the petiole, measured in lateral view from the posteriormost margin of the metapleural lobe to the posteriormost margin of the petiole.

**Petiole width (PW)**: Maximum width of petiole, measured in dorsal view.

**Postpetiole length (PPL)**: Maximum length of the postpetiole, measured in lateral view along the margin where postpetiolar tergite and sternite meet.

**Postpetiole width (PPW)**: Maximum width of postpetiole, measured in dorsal view.

**Scape index (SI)**: SL/HW × 100.

**Scape length (SL)**: Maximum length of the antennal scape, excluding the condylar bulb.

**Weber length (WL)**: Diagonal length of the mesosoma from the point at which the pronotum meets the cervical shield to the posterior base of the metapleuron, measured in lateral view.

## Results

### Asexual population

**Figure 1:**
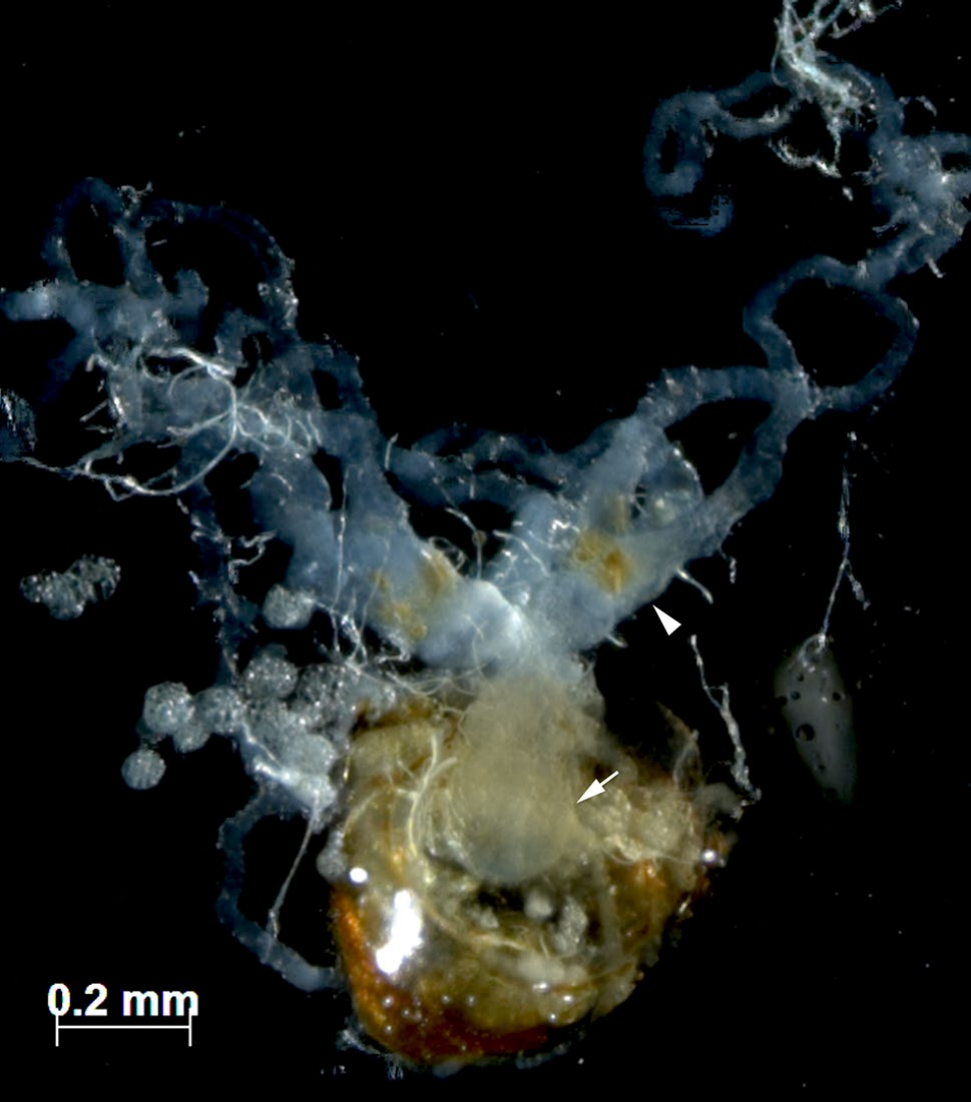
Reproductive organs of an asexually reproducing queen of the fungus-growing ant *Mycocepurus smithii*. The spermatheca is translucent indicating that it was empty (arrow), the ovaries are developed, and the presence of yellow bodies indicates that the queen was actively laying eggs (arrowhead).

Dissections revealed that the spermathecae of *M. smithii* queens from the Minas Gerais population were translucent, indicating that they were empty. The ovaries were fully developed and yellow bodies were present, indicating recent oviposition activity of the dissected queens (Fig. 1). In combination, the egg laying activity, the presence of *corpora lutea*, and the absence of sperm from the queens’ spermathecae demonstrate that the studied colonies from Minas Gerais reproduce thelytokously. In addition, laboratory colonies never produced males, which indirectly supports the absence of sexual reproduction in the Minas Gerais population.

**Figure 2:**
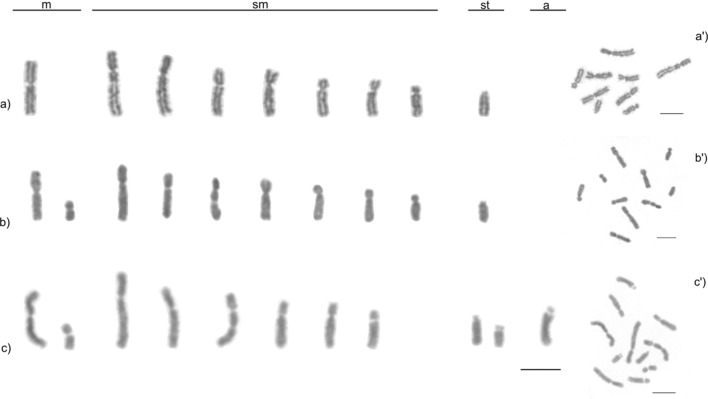
Karyotype and metaphase chromosomes, respectively, of asexual *Mycocepurus smithii* females organized according to their size and morphology*.* (**a**, **a′**) karyomorph 2n = 9 (1 m + 7sm + 1st); (**b**, **b′**) karyomorph 2n = 10 (2 m + 7sm + 1st); (**c**, **c′**) karyomorph 2n = 11 (2 m + 6sm + 2st + 1a). m metacentric, sm submetacentric, st subtelocentric, a acrocentric. Scale bar represents 5 µm.

**Figure 3:**
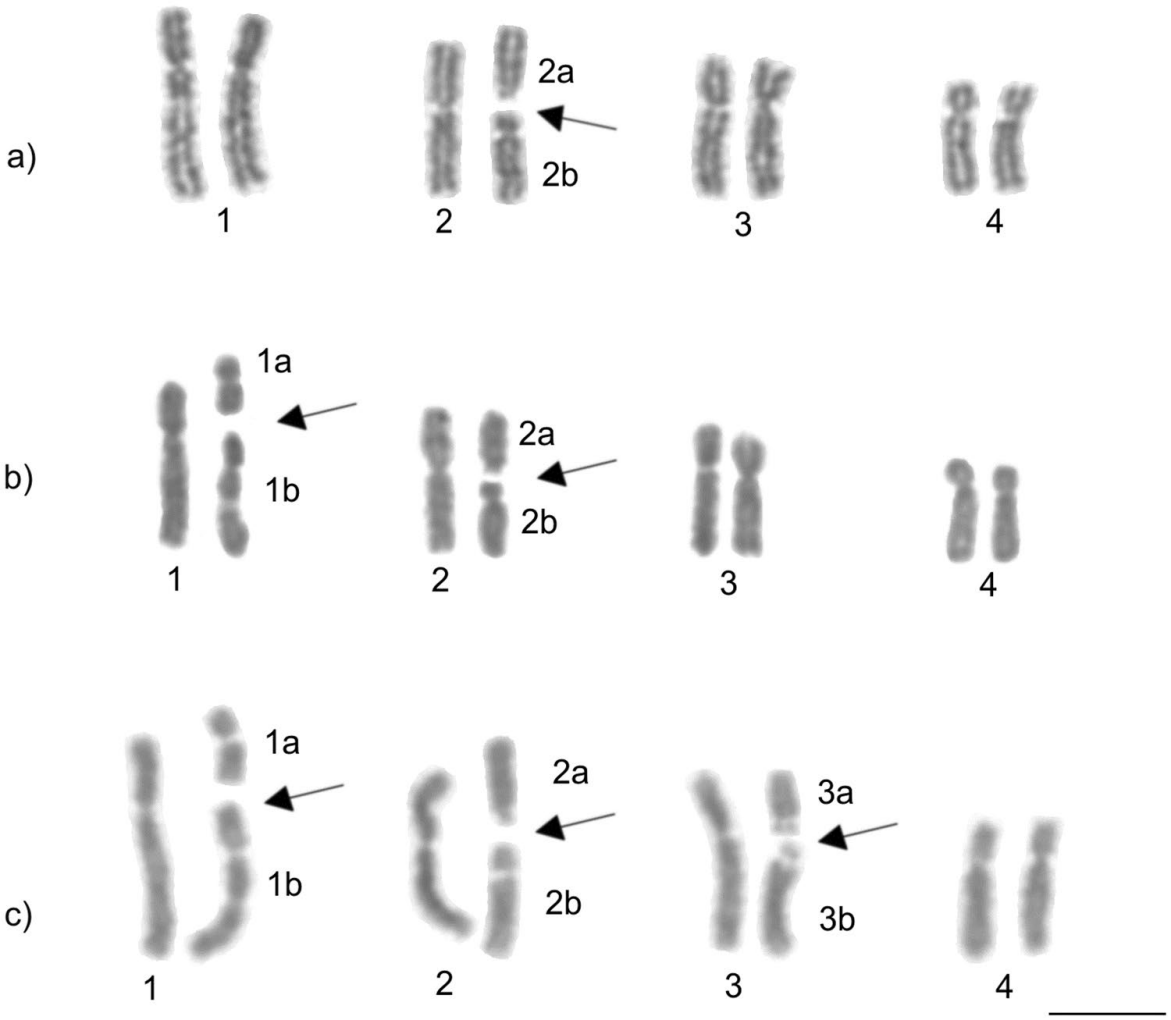
Female karyotypes of the asexual fungus-growing ant *Mycocepurus smithii* with probable pairings of karyomorphs: (**a**) 2n = 9 (1 m + 7sm + 1st), (**b**) 2n = 10 (2 m + 7sm + 1st), and (**c**) 2n = 11 (2 m + 6sm + 2st + 1a). Arrows indicate chromosomal rearrangements. The chromosomes were paired using numbers followed by letters to represent the possible pairings highlighting the increase of the decay of homologous chromosome pairs, especially in individuals with the karyomorph 2n = 11. Chromosome morphology: (**a**) metacentric chromosome: 2; submetacentrics: pair 1, 3, 4, and 2b; subtelocentric: 2a. (**b**) metacentrics: 1a, 2; submetacentrics: 1, 1b, 2b, pairs 3 and 4; subtelocentric: 2a. (**c**) metacentrics: 1a, 2; submetacentrics: 1, 1b, 2b, 3; subtelocentrics: 2a, 3a; acrocentric: 3b. Scale bar represents 5 µm.

Interestingly, the karyotypes of *M. smithii* females showed a variable number of chromosomes. Individuals from Ponte Nova, Minas Gerais had chromosome numbers of 2n = 9, 10, or 11, whereas the karyotypes of individuals from Viçosa, Minas Gerais had chromosome numbers of 2n = 10 or 11 (Table 1, Fig. 2). The assembly of karyotypes with the probable chromosome pairings is available in Fig. 3. The variation in chromosome number was only observed between different colonies, whereas the number and morphology of the chromosomes was maintained within the same colony. The chromosome morphology was also maintained among colonies with identical chromosome numbers.

**Figure 4:**
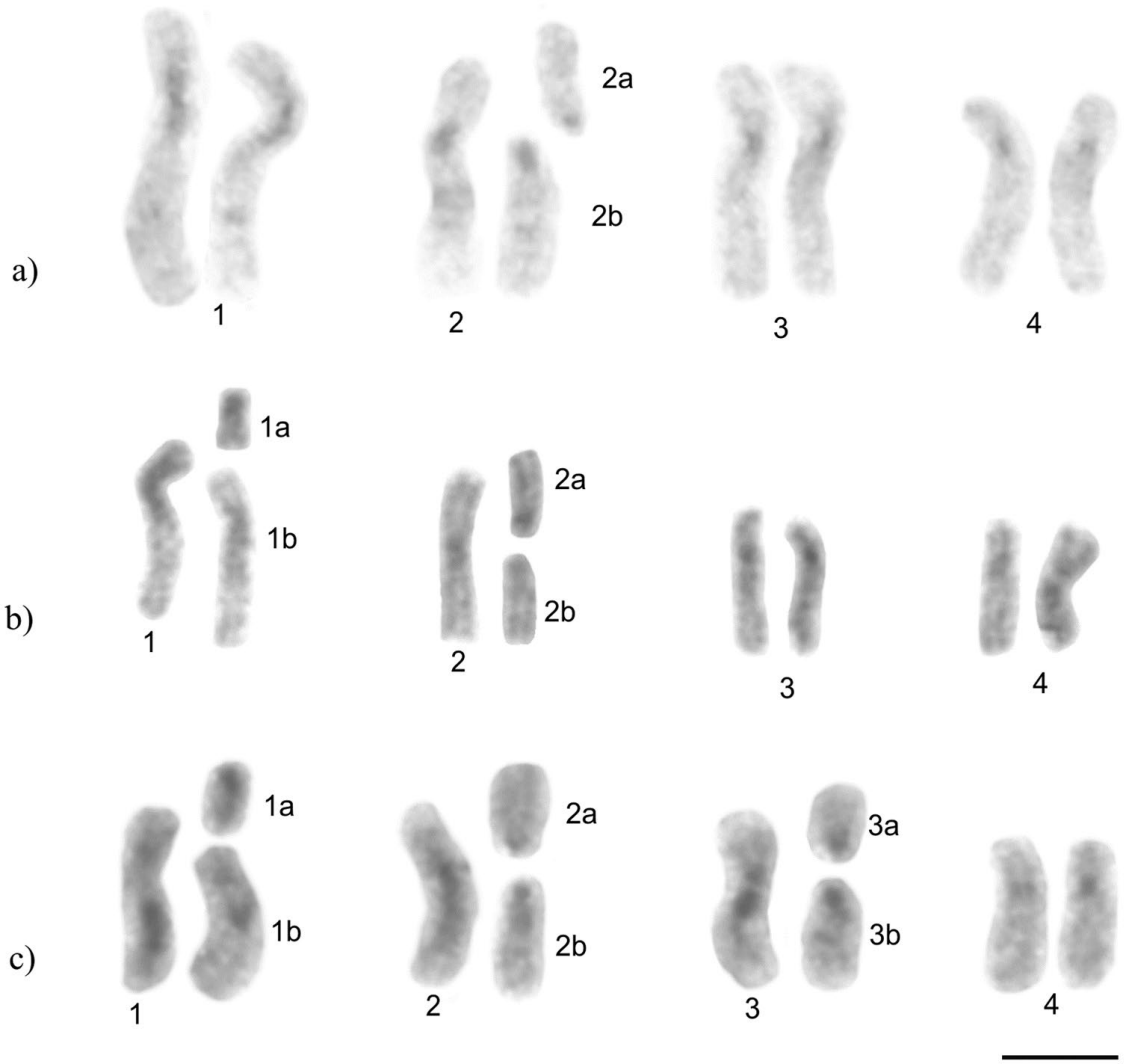
*Mycocepurus smithii* karyotypes from the asexual population marked with the C-banding technique for heterochromatin detection: (**a**) 2n = 9, (**b**) 2n = 10 and (**c**) 2n = 11. Scale bar represents 5 µm.

For the karyomorph 2n = 9 (Fig. 4a), heterochromatic bands were detected in centromeric regions of submetacentric chromosomes (pairs 1, 3, and 4) and in the single metacentric chromosome (pair 2). Additionally, interstitial bands were observed on the long arm of the largest submetacentric pair (pair 1), on the short arm of the subtelocentric (2a), and on the metacentric (2b) chromosomes. In the karyomorphs 2n = 10 (Fig. 4b) and 2n = 11 (Fig. 4c), the heterochromatin was located in centromeric regions of submetacentric (pairs 1, 3, and 4) and metacentric (pair 2) chromosomes. In addition, we also observed heterochromatin marks on the short arms of submetacentric (2b), metacentric (1a), subtelocentrics (2a, 3a), and acrocentric (3b) chromosomes, as well as interstitial bands on submetacentric chromosomes (1 and 1b).

**Figure 5:**
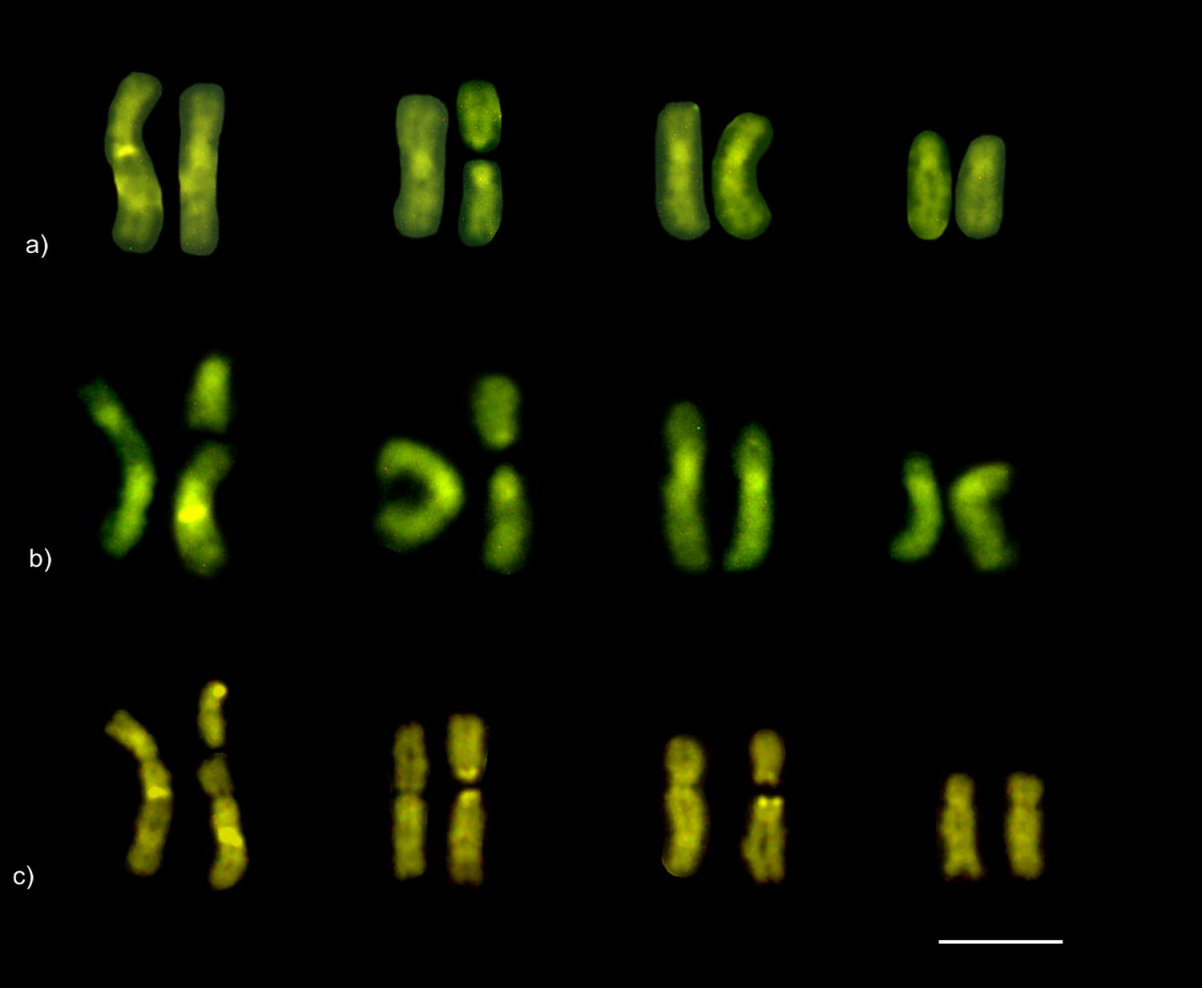
Karyotypes of *Mycocepurus smithii* from the asexual population stained with CMA_3_. (**a**) Karyomorph 2n = 9; (**b**) karyomorph 2n = 10; (**c**) karyomorph 2n = 11. Scale bar represents 5 µm.

The karyomorphs 2n = 9 (Fig. 5a), 2n = 10 (Fig. 5b), and 2n = 11 (Fig. 5c) showed multiple GC-rich regions (CMA_3_^+^), which were localized in the same chromosome regions as the heterochromatic bands. All the karyomorphs presented interstitial CMA_3_^+^ bands on the first submetacentric chromosome pair. AT-rich (DAPI^+^) regions were not observed.

**Figure 6:**
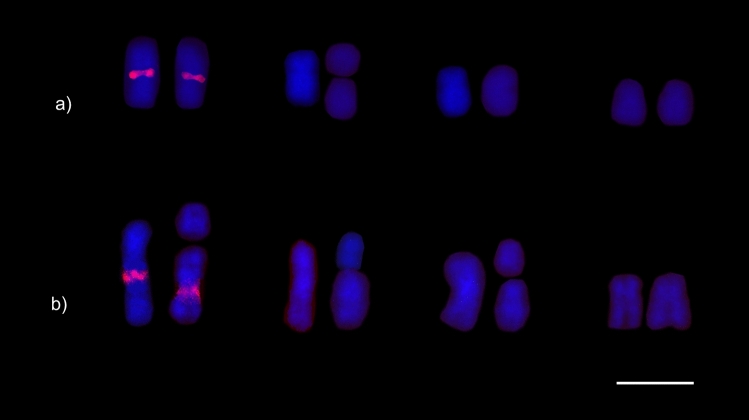
Karyotypes of *Mycocepurus smithii* from the asexual population marked via the FISH technique showing the presence of 18S rDNA genes (red regions) in two chromosomes for the karyomorphs (**a**) 2n =9  and (**b**) 2n = 11. Scale bar represents 5 µm.

The 18S rDNA gene clusters were located in the interstitial regions of the long arms of the largest submetacentric chromosome pair (pair 1) for the karyomorph 2n = 9 (Fig. 6a) and on the long arms of the submetacentric chromosomes (1 and 1b) for the karyomorph 2n = 11 (Fig. 6b). The region of the 18S rDNA genes colocalized with the CMA_3_^+^ interstitial markings on the chromosomes 1 and 1b. The presence of a clear secondary constriction that corresponded to the ribosomal genes and GC-rich regions was observed for both karyomorphs.

### Sexual population

**Figure 7:**
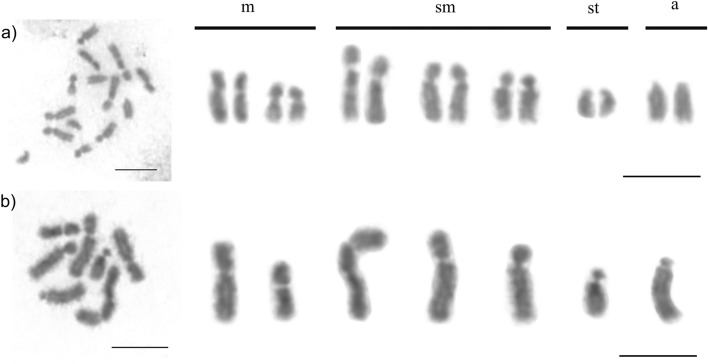
Metaphase chromosomes stained with Giemsa and the respective karyotypes from a sexual population of the fungus-growing ant *Mycocepurus smithii*. (**a**) Female (2n = 14) and (**b**) male (n = 7). m metacentric, sm submetacentric, st subtelocentric, a acrocentric. Scale bar represents 5 µm.

The chromosome number and karyotypic formula observed in the sexual population of *M. smithii* from Belém was uniform within the population with chromosome numbers of 2n = 14 (4m + 6sm + 2st + 2a) in females and n = 7 (2 m + 3sm + 1st + 1a) in males (Table 1, Fig. 7). Secondary constrictions were observed on the larger submetacentric chromosome pair using Giemsa and fluorochrome DAPI staining.

**Figure 8:**
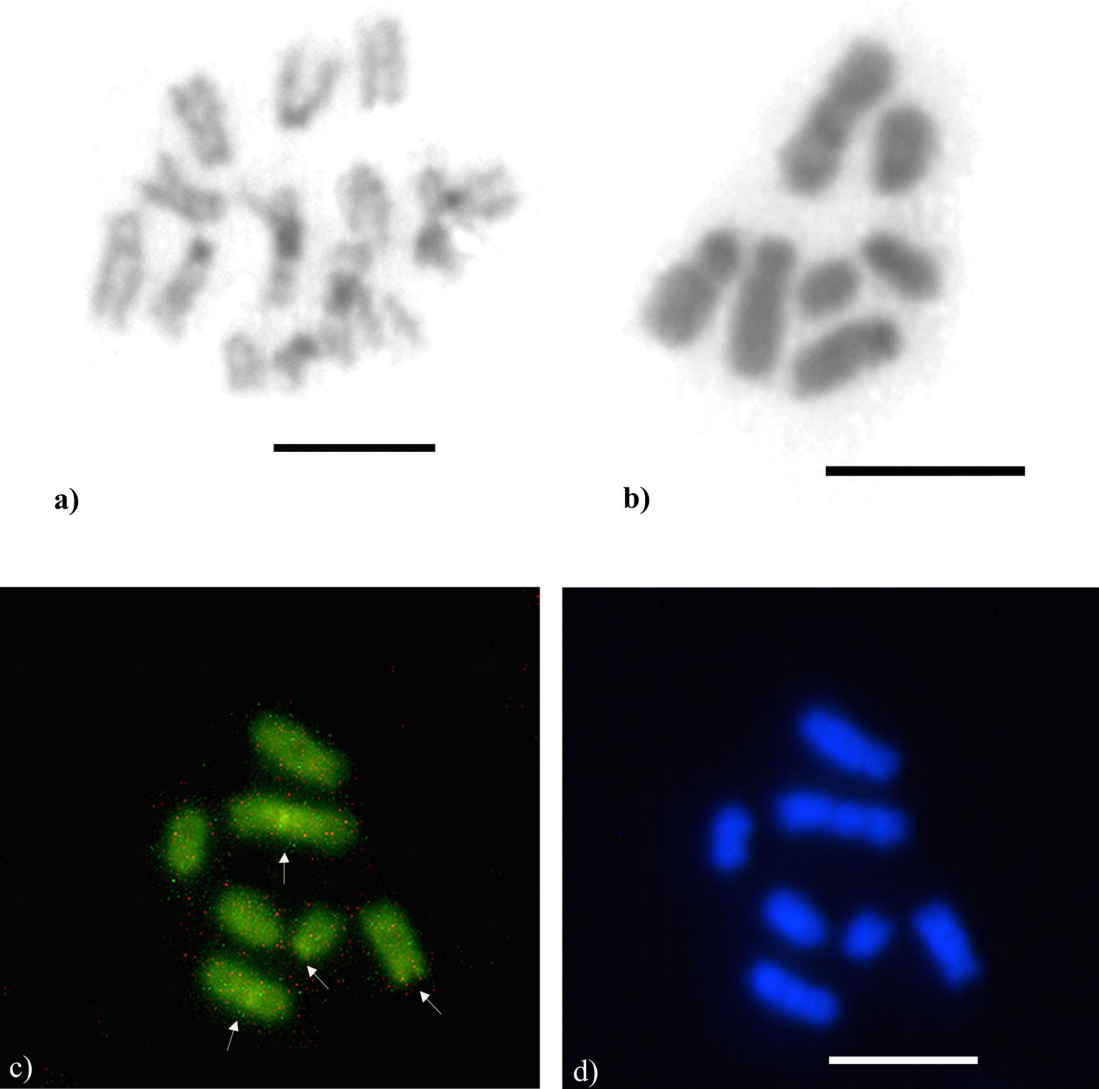
Metaphase chromosomes of the fungus-growing ant *Mycocepurus smithii* from a sexual population marked with the C-banding technique and stained with fluorochromes. (**a**) C-banding technique in female (2n = 14); (**b**) male (n = 7). Black regions indicate the presence of heterochromatin. (**c**) Metaphase stained sequentially with the fluorochromes CMA_3_; (**d**) DAPI, for detection of regions rich in GC and AT base pairs, respectively. White arrows indicate the presence of GC-rich blocks. Scale bar represents 5 µm.

**Figure 9:**
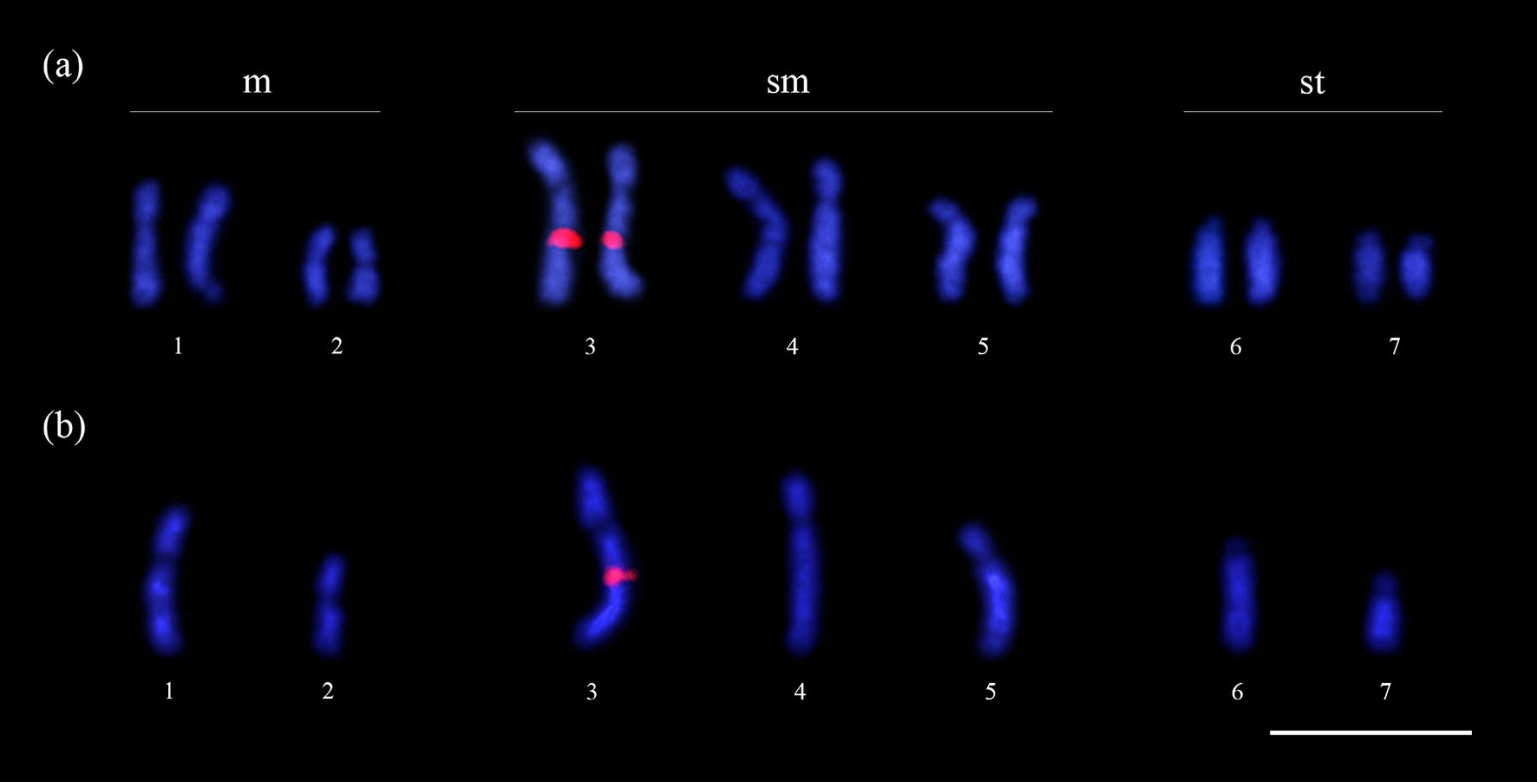
Metaphase chromosomes of *Mycocepurus smithii* from the sexual population stained with the FISH technique showing the presence of 18S rDNA genes (red regions) in (**a**) female (2n = 14) and (**b**) male (n = 7). Scale bar represents 5 µm.

Heterochromatic blocks were observed on short arms of some chromosomes (2nd and 3rd submetacentric and the subtelocentric pair) and strong interstitial markings were also observed on the largest submetacentric chromosome pair (Fig. 8a,b). GC-rich regions were visualized on the short chromosome arms and intrachromosomal regions that colocalized with heterochromatin blocks. In addition, we observed an interstitial band on the long arm of the largest submetacentric chromosome pair (Fig. 8c), which colocalized with 18S rDNA gene clusters (Fig. 9). AT-rich regions with differential staining with DAPI were not observed, therefore AT-rich regions were observed in uniform patterns (Fig. 8d). Only negative regions, complementary to GC-rich regions, were detected.

### Diagnosis of *Mycocepurus smithii* males

#### Male diagnosis

**Figure 10:**
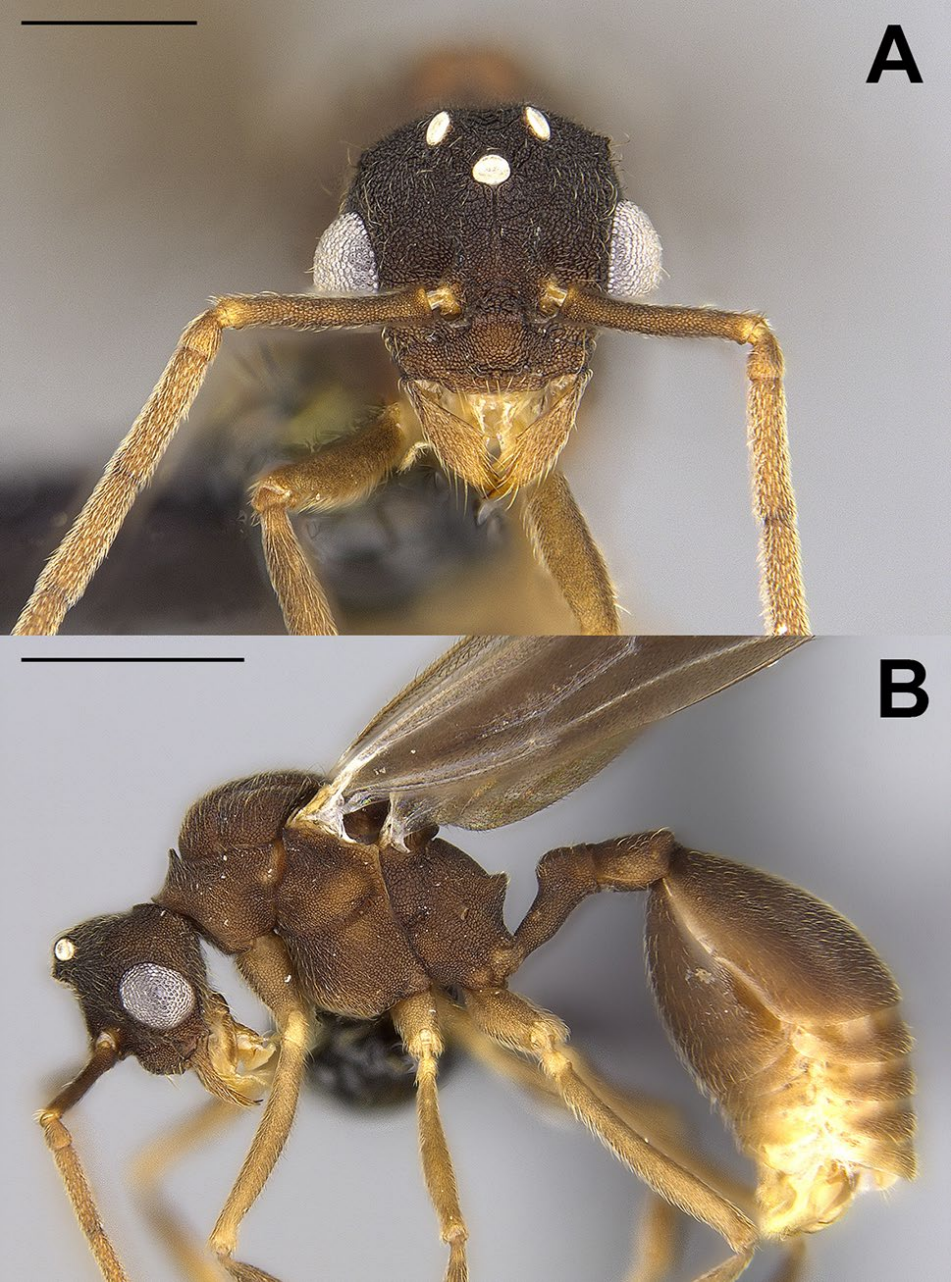
Males of the fungus-growing ant *Mycocepurus smithii* in full-face (**a**) and lateral (**b**) views. The scale bars represent 0.5 mm in (**a**) and 1 mm in (**b**). Male individuals were collected from a sexually reproducing colony in Belém, Pará in Brazil.

Measurements (n = 5). HW 0.72–0.73, HL 0.66–0.69, SL 0.5–0.53, WL 1.34–1.39, PPW 0.36–0.41, PW 0.17–0.19, PL 0.42–0.44, PPL 0.25–0.31, EL 0.3, CI 107–109, SI 68–74. Medium-size *Mycocepurus* male (WL 1.34–1.39) with relatively long appendages (Fig. 10). Body surface opaque, medium to light brown, densely and finely reticulate-punctate; appendages light to yellowish-brown. Pilosity of body surface appressed. Dorsal and lateral surfaces of mesosoma and dorsal surface of head with weakly pronounced rugae. Antennae 13-segmented with short scapes (SI 68–74). Funiculus long with first funicular segment only slightly longer than wide, funicular segments 2–11 approximately 2.5 × longer than wide. In full face view, head shape trapezoidal, wider than long (CI 107–110), occipital corners with minute denticles. Eyes large, convex (EL 0.3). Ocelli slightly elevated above posterior margin of head, all ocelli of equal size. Clypeus broadly triangular, conically elevated between antennal insertions, with long median seta. Mandibles elongate, triangular, with three teeth, including a tiny basal and a large, broad apical tooth. Maxillary palps 4-segmented, labial palps 2-segmented. Anterior lateral pronotal spines reduced to tiny denticles. Scutellum posteriorly bidentate. Propodeal spines small, approximately as long as wide at base, forming broadly triangular spines. Petiole with long anterior peduncle. Postpetiole trapezoidal in shape, approximately 1.3× wider than long, rounded, dorso-ventrally flattened. First gastral segment large, convex, almost 1.5× longer than wide. Genitalia projecting forward from tip of metasoma. Wings infuscated, venation as described in Kempf’s generic diagnosis^36^.

#### Material examined

BRAZIL: Pará, Belém, Campus of Universidade Federal Rural da Amazônia (UFRA), 01.4500° S, 048.4333° W, elevation 10 m above sea level, 11 October 2012, col. L. A. C. Barros, H. J. A. C. de Aguiar; acc. no. Lu 136.

#### Discussion

Currently, the males of only three *Mycocepurus* species have been diagnosed including *M. goeldii*^36,37^, *M. obsoletus *^20,36^, and *M. castrator*^38^. The male of *M. smithii* can be readily distinguished from the male of *M. goeldii* by its significantly smaller size, the lighter coloration, the lack of deep rugulae on the mesosoma, and the highly reduced anterior lateral pronotal spines. Compared to *M. goeldii* and in relation to body size, the *M. smithii* males also exhibit shorter antennal scapes, shorter funicular segments 2–11, and a longer petiolar peduncle.

The males of *M. obsoletus* were first collected in Rio Claro, São Paulo in Brazil during a mating flight^39^. Kempf^36^ suggested that these individuals could represent the male sex of *M. smithii* and diagnosed the individuals in his taxonomic revision of the genus. Almost 50 years later, the males of *M. obsoletus* were collected in association with workers near Brasília, revealing that the putative *M. smithii* males described by Kempf represented in fact the males of *M. obsoletus*^20^. The male of *M. smithii* is similar in size to the male of *M. obsoletus*, but can be distinguished by the lighter coloration, the longer mandibles, the reduced number of mandibular teeth, the more acute propodeal spine, and the longer petiolar peduncle.

*Mycocepurus castrator* is an inquiline social parasite of *M. goeldii* and due to its gynaecomorphism the *M. castrator* males represent atypical *Mycocepurus* males that cannot be confused with any other male in the genus^38^.

### Diagnosis of *M. smithii* spermatozoa

**Figure 11:**
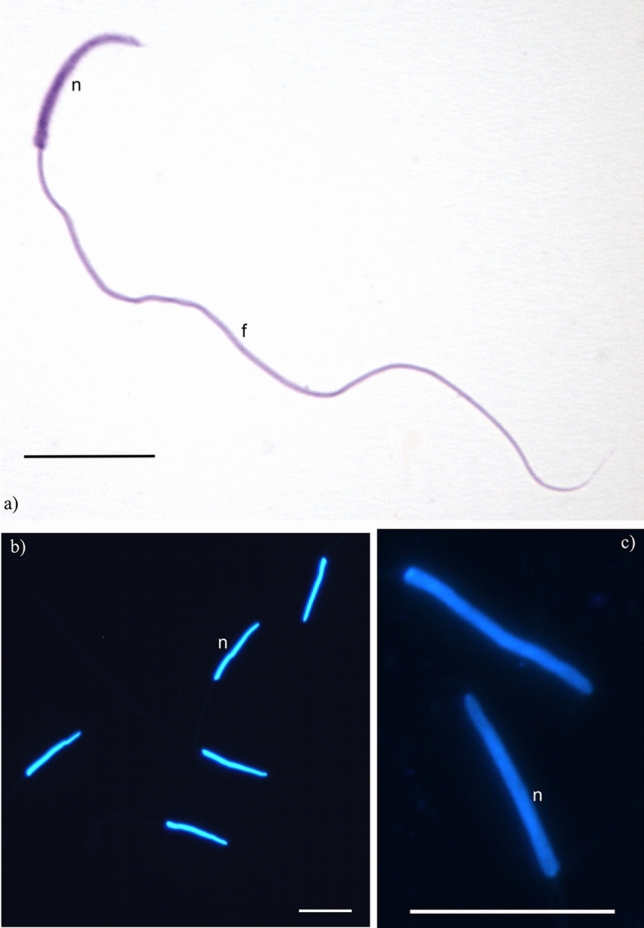
Morphology of the sperm of the fungus-growing ant *Mycocepurus smithii*. (**a**) Sperm stained with Giemsa; (**b**, **c**) nuclei stained with DAPI. Nucleus (n), flagellum (f). Scale bar represents 10 µm.

The spermatozoa of *M. smithii* are long, threadlike, and linear (Fig. 11) with a total length of 69.03 µm (± 3.1) and a nucleus length of 12.2 µm (± 0.64). The nuclei have a uniform shape and taper abruptly at the anterior end. The head and the flagellum of the spermatozoa are clearly distinct (Fig. 11).

## Discussion

### Karyotypes in asexually reproducing *M. smithii*

Our comparative cytogenetic study of asexually and sexually reproducing *M. smithii* colonies reveals that in an asexual population from Minas Gerais karyotypes were constant within *M. smithii* colonies but variable between colonies. We identified intraspecific variation between sympatric *M. smithii* colonies with three distinct karyomorphs where the number of diploid chromosomes equaled 9, 10, and 11. In addition, the karyomorphs differed not only in their numbers but also in the morphology of the chromosomes. Although the chromosome numbers were constant within the same colonies, the described variation was observed between colonies that were located in the same population.

Upon examining the first sample, we first believed that the karyomorph 2n = 11 was a haploid karyotype because it presented an uneven chromosome number and almost all the chromosomes were unpaired. Closer study revealed that this karyotype belonged to female individuals, which are expected to be diploid in arrhenotokous Hymenoptera. However, studying the prepupae and larvae of queens confirmed that the observed individuals were in fact females due to the absence of testes.

### Karyotypes in sexually reproducing *M. smithii*

In contrast to the asexual individuals, the karyotypes of *M. smithii* females from sexually reproducing colonies were constant*.* The worker and gyne larvae from the sexually reproducing *M. smithii* population in Belém, Pará had identical diploid chromosome numbers (2n = 14), whereas male karyotypes were characterized by half the chromosome number (n = 7), which is expected for haploid males produced via arrhenotokous parthenogenesis. Female karyotypes also exhibited the proper homologous chromosome pairings, which is consistent with female karyotypes reported for most sexually reproducing ant species^40,41^. In addition, variation in chromosome number was not observed between individuals of different sexually reproducing *M. smithii* colonies, which contrasts with our observation in the asexual population.

### Chromosome decay and heterozygosity in unpaired chromosomes

The karyotype differences observed between asexual and sexual *M. smithii* populations, as well as within the asexual *M. smithii* population, were characterized by numerical and morphological differences probably resulting from centric fission and other chromosome rearrangements. In the absence of meiosis, centric fission can increase the chromosome number, whereas further minor and independently occurring rearrangements on both the rearranged chromosome and the larger ancestral chromosome removes traces of homology between them, leading to the decay of the diploid chromosome structure. The observed karyotypes in asexual *M. smithii* populations were consistent with this expectation because the karyomorph 2n = 9 was characterized by three pairs of homologous chromosomes, whereas the karyomorph 2n = 10 presented only two homologous chromosome pairings, and the karyotype 2n = 11 showed only a single set of properly paired homologous chromosomes. The loss of homologous chromosome pairings is indicative of a decay in the diploid structure of the individual karyotypes, and the most extreme form of decay was observed in karyotype 2n = 11. In contrast, a decay of homologous chromosome pairs could not be observed in the sexually reproducing population of *M. smithii*, and in that population the chromosomes were perfectly paired with a haploid chromosome number of n = 7 and a diploid set of 2n = 14.

Our results indicate decaying karyotypes in an asexual *M. smithii* population, which is consistent with the hypothesis that natural selection is relaxed in the absence of meiosis^5,42^. Under those circumstances, chromosomes accumulate structural rearrangements and can become heterozygous in the absence of forced pairings during meiosis^5,7^, which has also been discussed as a potential mechanism for sexual chromosome diferentiation^43,44^. Ultimately, the accumulation of heterozygosity on individual chromosomes should lead to the loss of homologous chromosome pairs. Our results are consistent with this prediction because in asexual *M. smithii* population, we observe the partial decay of homologous chromosome structure, whereas chromosome structure is preserved in a sexually reproducing *M. smithii* population in the face of meiosis and genetic recombination.

*Mycocepurus smithii* constitutes of a mosaic of asexual and sexual populations and the loss of sexual reproduction likely evolved repeatedly and independently from the ancestral sexual population^21^. Because asexual populations likely evolved convergently, we hypothesize that additional chromosomal variations will be recognized in other asexual populations. In contrast, we would expect that the karyotypes in sexual *M. smithii* populations are uniform. Among the different asexual colonies from Minas Gerais bearing karyomorphs of 2n = 10 and 2n = 11, variation in chromosomal morphology was not observed, suggesting a single origin of these karyomorphs in our study population (Table 1).

Cytogenetic data is available for at most three *Mycocepurus* species including *M. goeldii* (2n = 8)^45^, *Mycocepurus* sp. (2n = 8)^46^, and the asexual (2n = 9, 10, 11) and sexual (2n = 14, n = 7) populations of *M. smithii* studied here. Considering that cytogenetic data is available for two sexually reproducing species with a karyotype of 2n = 8, we initially expected that the asexual karyotype would be 2n = 8. The asexual karyomorph of 2n = 9 represents the smallest amount of decay in diploid chromosome structure. The observed diploid chromosome number of 2n = 14 in the sexual *M. smithii* population is the highest number of chromosomes observed in *Mycocepurus* ants so far.

Chromosome rearrangements were previously reported for ants such as in sexually reproducing bulldog ants in the *Myrmecia pilosula* species complex^25,47^. Interestingly, variation in chromosome number was reported from individuals belonging to the same colony even when only few individuals were sampled. Imai and colleagues^25,47^ suggested that the observed variation in chromosome number was a consequence of variable chromosome numbers in the parental generation, which is different from the variation observed in the asexual *M. smithii* where variation is likely caused by the decay of chromosome structure due to the lack of meiosis and where variation occurred between but not within colonies.

Outside the Hymenoptera, cyclic parthenogenesis is observed in aphids where sexually and asexually reproducing generations alternate with each other. In contrast, many members of the aphid Tribe Tramini reproduce exclusively asexually^48^, and a decay of karyotype diploidy with a high degree of chromosome diversification can also be observed in these species.

### Cytogenetic data from other thelytokous ant species

Among ants, thelytokous parthenogenesis has been documented for at least 20 species in four subfamilies including the Dorylinae, Formicinae, Myrmicinae, and Ponerinae^10,12–15^. Cytogenetic information is available for six thelytokous ant species including *Ooceraea biroi* (Forel, 1907)*, Paratrechina longicornis* (Latreille, 1802), *Platythyrea punctata* (Smith, 1858), *Pristomyrmex punctatus* (Smith, 1860), *Vollenhovia emeryi* Wheeler, 1906, and *Wasmannia auropunctata* (Roger, 1863)^40,49–55^. In contrast to the results obtained here for *M. smithii*, all of the abovementioned thelytokous ant species maintain their chromosome numbers and their homologous chromosome pairings^40^. In addition, all of these species are characterized by regular or occasional male production via arrhenotoky, except for *P. longicornis* where males are clones of their fathers^56^, suggesting that meiosis is still functional in those species^10^. In contrast, the production of males was exclusively observed in sexually reproducing *M. smithii* populations, and males do not seem to be produced in asexually reproducing *M. smithii* colonies suggesting that meiosis may be dysfunctional in these asexual populations.

### Heterochromatin distribution pattern

Heterochromatin plays an important architectural role in chromosome structure and it is enriched in tandem repetitive sequences. The heterochromatin distribution on chromosomes is related to specific functions. For example, centromeric heterochromatin is important for the accurate segregation of chromosomes^57^. In addition, other regions with accumulations of highly repetitive DNA can be observed throughout the genome extending beyond the centromere. Heterochromatin can influence the frequency of structural rearrangements in neighboring regions^58^, and consequently, it is essential for chromosome stability^47^. According to the Minimum Interaction Theory (MIT) proposed by Imai and colleagues^47^, chromosome fissions are the principal rearrangements in the karyotype evolution of ants because they reduce chromosome sizes and thus reduce deleterious chromosome interactions in the interphase nucleus. The posterior heterochromatin growth after fission plays an important role in allowing telomeric maintenance and chromosome stability, yielding chromosomes with heterochromatic arms.

The repetitive DNA sequences that constitute the heterochromatin in fungus-growing ant chromosomes, including *M. goeldii*, show richness of GC-base pairs, which is an uncommon trait in other ant species. It was suggested that this GC-rich heterochromatin originated in the common ancestor of the fungus-growing ants, but this hypothesis requires further study^41,45,59^. Notwithstanding, the results obtained in this study demonstrate that both asexual and sexual populations of *M. smithii* also possess a GC-rich heterochromatin composition similar to *M. goeldii*, which adds to the number of attine species with this characteristic trait and strengthens the hypothesis of a common origin of GC-rich heterochromatin in fungus-growing ants.

The heterochromatin distribution pattern on chromosomes aides to recognize possible chromosomal pairs in asexual *M. smithii* individuals. In addition, it strongly suggests the occurrence of centric fission in *M. smithii* according to Imai’s Minimum Interaction Theory (MIT)^47^ because we observed heterochromatin on short arms of rearranged chromosomes in sexual (2n = 14) and in asexual populations (2n = 9, 10, 11). In addition, the karyomorph 2n = 9, i.e., the karyotype with less chromosomal decay, presented heterochromatin on centromeric or pericentromeric regions of all the chromosomes and this pattern is similar to the one observed in *M. goeldii* (2n = 8)^45^. Thus, the information about heterochromatin distribution on the chromosomes provides important insights into the pathways of karyotype evolution in ants^27,47^.

### 18S rDNA gene distribution patterns

Ribosomal genes are important chromosomal markers used in different organisms, including ants, because these genes follow specific patterns of chromosomal organization. A single chromosome pair bearing all the rDNA genes is the most common and plesiomorphic pattern observed in diploid karyotypes of ants^60^. In *M. smithii*, 18S rDNA genes were located on a single chromosome pair in asexual individuals with the karyomorphs 2n = 9 (chromosome pair 1) and 2n = 11 (chromosomes 1 and 1b), as well as in the sexual individuals with the karyomorph 2n = 14 (chromosome pair 3). In *M. smithii* males the rDNA was located on a single chromosome (chromosome 3). These data corroborate our findings that asexual individuals are indeed diploid. In asexual individuals with the karyomorph 2n = 11, the homologous chromosomes showed the same morphology (submetacentric), but we also observed a size difference between them suggesting the occurrence of centric fission in the rDNA-bearing chromosome pair.

Both asexual and sexual populations had a secondary constriction in at least one of the homologous chromosomes that were colocalized with the 18S rDNA clusters and GC-rich heterochromatic bands. According to Teixeira et al.^60^, the presence of single rDNA sites on the chromosomes is influenced by their position on the chromosome because intrachromosomal regions are less prone to rearrangements compared to terminal regions. The analysis of rDNA clusters in further asexual populations should be promising to better understand the selective pressures on rDNA clusters in the genome.

A prior population genetic study of *M. smithii* suggested that individuals in asexual populations were genetically identical across multiple generations and that males and sexual recombination were absent from asexual populations^21^. The strict clonality of asexual *M. smithii* populations suggested either apomixis or automixis with central fusion, i.e., a form of asexual reproduction with low recombination rates, as the cytological mechanism underlying thelytokous parthenogenesis in *M. smithii*^21^. Our cytogenetic results are consistent with this general interpretation and lend further support to the apomixis hypothesis because the karyotypes of asexual *M. smithii* individuals show that heterozygous chromosome configurations likely resulted from chromosome rearrangements in the absence of meiosis. In contrast, the presence of aneuploid individuals would strengthen the hypothesis that asexual *M. smithii* reproduce via automixis with central fusion, however, such individuals were not detected. Improbable and unpaired chromosomal rearrangements are not expected to be maintained in the absence of meiosis in eukaryotic genomes and its loss would allow for the decay of diploid structures, as observed in asexual *M. smithii* individuals.

### Males of *M. smithii*

*Mycocepurus smithii* reproduces via thelytokous parthenogenesis and only few populations in the center of the species’ distribution range in the Brazilian Amazonas region are known to reproduce sexually^21^. Previously, the existence of *M. smithii* males was inferred from the haploid sperm extracted from the spermathecae of *M. smithii* queens^21^. The male individuals described here finally provide evidence that recombinant *M. smithii* workers are the result of syngamy resulting from *M. smithii* males inseminating queens of the same species. The putative males of *M. smithii* from Rio Claro, São Paulo^36^ were previously identified as males of *M. obsoletus*^20^, and the morphological comparison between males of *M. smithii* and *M. obsoletus* could further identify morphological characters that are diagnostic of each species.

The comparative study of spermatozoa is useful for understanding the reproductive biology of ants in more detail and to obtain additional morphological characteristics that could be used for comparative studies of closely related species. The sperm length in ants varies from 53 µm in *Pseudomyrmex termitarius* (Smith, 1855)^61^ to 230 µm in *Apterostigma* fungus-growing ants^62^. Information related to spermatozoa in ants is still scarce^62,63^. Nevertheless, comparing the structure of these cells can be insightful when characterizing a species and solving taxonomic problems of cryptic species existing in sympatry, such as *Neopoera inversa* (Smith, 1858) and *Neoponera villosa* (Fabricius, 1804) that showed species specific differences in the length of the sperm nucleus^64^.

The spermatozoa of attine ants are highly variable in length with 67.06 µm in *Atta sexdens* (Linnaeus, 1758), being the smallest known sperm cell in the fungus-growing ants, to 230.49 µm in an unidentified *Apterostigma* species, being the longest known sperm cell in fungus-growing ants^62^. With a total length of 69 µm, the spermatozoa of *M. smithii* are among the smallest observed in the fungus-growing ants, similar in length to spermatozoa of *Atta sexdens*. In general, in the lower attine ants spermatozoa length decreases with the increase of colony size for the queens that copulate with a single male, which suggests that the production and storage of sperm affect the evolution of sperm length^62^. The genus *Apterostigma* is a member of the paleoattine fungus-growing ants which also includes *Mycocepurus* and *Myrmicocrypta*^65,66^, and the size difference of sperm length between different species of *Apterostigma* ants is remarkable, ranging from 138.06 to 230.49 µm^62^.

The nucleus length of *M. smithii* spermatozoa measures 12.2 (± 06.4) µm. The nucleus length in other ant species ranges from 9 to 50 µm in *Dolichoderus* and *Nesomyrmex*, respectively^63^. The available data for fungus-growing ant sperm refers exclusively to the total length, preventing further comparisons.

## Conclusions

Cytogenetic studies are important for revealing the cytological mechanisms and consequences underlying the evolution of asexual reproduction in ants^67^. To the best of our knowledge, we here report the first cytogenetic analysis for ants that demonstrates chromosome number variation within a species that reproduces via thelytokous parthenogenesis. These chromosomal data obtained for the asexual population of *M. smithii* corroborate the decay of diploid chromosome structure in the absence of meiosis due to relaxed natural selection on chromosome architecture. Comparative cytogenetic data obtained for both sexual and asexual populations of *M. smithii* indicate that centric fission plays a role in the origin of chromosomal variation observed in *M. smithii*, which is consistent with Imai’s Minimum Interaction Theory^47^. The maintenance of unpaired chromosomal homologs in the karyotypes of asexual *M. smithii* individuals further supports the hypothesis that apomixis is the cytological mechanism underlying thelytoky in *M. smithii*, but automixis with central fusion cannot be ruled out. Our study supports the hypothesis that asexual reproduction becomes obligate in *M. smithii* populations once the ability to reproduce sexually is lost and that asexually reproducing individuals are unlikely to revert to sexual reproduction.

Furthermore, we confirm the existence of sexually reproducing *M. smithii* populations in the Amazonas region of Brazil. We describe the first karyotypes of males and females from a sexually reproducing population, contributing to the knowledge about chromosomal architecture in the species. Furthermore, we provide the first taxonomic diagnosis of the *M. smithii* males and describe the structure of their spermatozoa.

In summary, our comparative cytogenetic study demonstrates the rapid decay of chromosome architecture in the absence of meiosis and genetic recombination, contributing to our understanding about the evolution and the cytological mechanisms associated with thelytokous parthenogenesis in fungus-growing ants.
